# The prevalence of imposter syndrome among neurosurgeons in Europe: An EANS YNC survey

**DOI:** 10.1016/j.bas.2024.102816

**Published:** 2024-04-16

**Authors:** Cesare Zoia, Martin N. Stienen, Ismail Zaed, Grazia Menna, Cristina C. Aldea, Jiri Bartek, Marlies Bauer, Diogo Belo, Evangelos Drosos, Christian F. Freyschlag, Stanislav Kaprovoy, Milan Lepic, Laura Lippa, Malte Mohme, Stefan Motov, Michael Schwake, Toma Spiriev, Felix C. Stengel, Fabio Torregrossa, Giovanni Raffa, Maria L. Gandía-Gonzalez

**Affiliations:** a Neurosurgery Unit, Ospedale Moriggia Pelascini, Gravedona e Uniti, Italy; b Department of Neurosurgery and Spine Center of Eastern Switzerland, Cantonal Hospital, St.Gallen, St.Gallen, Switzerland; c Department of Neurosurgery, Neurocenter of the Southern Switzerland, Regional Hospital of Lugano, Ente Ospedaliero Cantonale, Lugano, Switzerland; d Department of Neurosurgery, A. Gemelli University Hospital Foundation IRCCS, Catholic University of the Sacred Heart, Rome, Italy; e Department of Neurosurgery, Cluj County Emergency Hospital, University of Medicine and Pharmacy Iuliu Hatieganu, Cluj-Napoca, Romania; f Department of Clinical Neuroscience, Karolinska Institutet and Department of Neurosurgery, Karolinska University Hospital, Stockholm, Sweden & Department of Neurosurgery, Rigshospitalet, Copenhagen, Denmark; g Department of Neurosurgery, Medical University of Innsbruck, Innsbruck, Austria; h Neurosurgery Department, Centro Hospitalar Lisboa Norte (CHLN), Lisbon, Portugal; i Salfort Royal NHS Foundation Trust, Manchester, United Kingdom; j Burdenko Neurosurgical Center, Department of Spinal and Peripheral Nerve Surgery, Department of International Affairs, Moscow, Russia; k Clinic for Neurosurgery, Military Medical Academy, Belgrade, Serbia; l Department of Neurosurgery, ASST Ospedale Niguarda, Milano, Italy; m Department of Neurosurgery, University Medical Center Hamburg-Eppendorf, Hamburg, Germany; n Department of Neurosurgery, University Hospital Muenster, Germany; o Department of Neurosurgery, Acibadem CityClinic University Hospital Tokuda, Sofia, Bulgaria; p Department of Neurologic Surgery, Mayo Clinic, Rochester, MN, USA; q Department of Neurosurgery and Department of Otolaryngology - Head and Neck Surgery, Mayo Clinic Rhoton Neurosurgery and Otolaryngology Surgical Anatomy Program, Rochester, MN, USA; r Neurosurgical Unit, Department of Biomedicine, Neurosciences and Advanced Diagnostics (BiND), University of Palermo, Palermo, Italy; s Division of Neurosurgery, BIOMORF Department, University of Messina, Messina, Italy; t Department of Neurosurgery, Hospital Universitario La Paz, Madrid, Spain

**Keywords:** Imposter syndrome, Residency, Neurosurgery, Burnout syndrome, Imposterism

## Abstract

**Introduction:**

Imposter syndrome (IS), characterized by persistent doubts about one's abilities and fear of exposure as a fraud, is a prevalent psychological condition, particularly impacting physicians. In neurosurgery, known for its competitiveness and demands, the prevalence of IS remains high.

**Research question:**

Recognizing the limited literature on IS within the neurosurgical community, this European survey aimed to determine its prevalence among young neurosurgeons and identify associated factors.

**Material and methods:**

The survey, conducted by the Young Neurosurgeon Committee of the European Association of Neurosurgical Societies, gathered responses from 232 participants. The survey included demographics, the Clance Imposter Phenomenon Survey (CIPS), and an analysis of potential compensatory mechanisms.

**Results:**

Nearly 94% of respondents exhibited signs of IS, with the majority experiencing moderate (36.21%) or frequent (40.52%) symptoms. Analyses revealed associations between IS and factors such as level of experience, sex, and board-certification.

**Discussion and conclusion:**

The findings suggest a significant prevalence of IS among young neurosurgeons, with notable associations with sex and level of experience. Compensatory mechanisms, such as working hours, article reading, and participation in events, did not show significant correlations with IS. Notably, male sex emerged as an independent protective factor against frequent/intense IS, while reading more than five articles per week was identified as a risk factor. The identification of protective and risk factors, particularly the influence of gender and reading habits, contributes valuable insights for developing targeted interventions to mitigate IS and improve the well-being of neurosurgeons.

## Highlights

• Imposter Syndrome is highly prevalent among young neurosurgeons in Europe.
• Among those exhibiting Imposter Syndrome, the majority experience moderate or frequent symptoms.
• There are associations between Imposter Syndrome and level of experience, sex, and board-certification.
• Working hours, article reading, and participation in events, did not show significant correlations with Imposter Syndrome.
• Male sex emerges as an independent protective factor against frequent/intense Imposter Syndrome.

## 1 Introduction

Imposter, or impostor, syndrome (IS) has been firstly described by two psychologists, Clance and Imes, in their landmark paper published in 1978 (Clance and Imes, 1978). Nowadays, IS is defined as a psychological condition in which people doubt their skills, talents, or accomplishments and have a persistent internalized fear of being exposed as frauds (Langford and Clance, 1993).

Since those first studies, a number of subsequent reports have identified relatively high prevalence among physicians, regardless of ethnicity and age group. Bravata et al. consider IS responsible for an increased rate of burnout, depression, and anxiety (Bravata et al., 2020). Given the importance of the topic and its multiple social implications, IS has become a widely addressed topic in the literature. According to Siddiqui and colleagues, Impostor Phenomenon (IP) has been documented in numerous high-achieving fields. Multiple studies have linked IP with conditions like depression, anxiety, burnout, and perfectionism. The potential ramifications of IP within the healthcare industry are worrisome, highlighting the urgent necessity to confront and minimize its effects within this sector (Siddiqui et al., 2024). This is especially concerning in medical specialties, such as neurosurgery, known for being one of the most competitive, and both physically and mentally demanding fields. A first recent survey among the Italian neurosurgical community defined the prevalence rate of IS to be as high as 81.6% (Zaed et al., 2022).

Given the paucity of scientific literature on work stress and IS within the neurosurgical community, the authors developed this European survey with the primary aim to define the prevalence of IS among young neurosurgeons in European countries, and, as the secondary aim, to identify factors associated with IS.

## 2 Material and method

### 2.1 Survey design

This survey was designed to assess the prevalence of IS among young neurosurgeons and neurosurgical trainees in Europe. Survey items were adapted from the previously published survey about imposterism in young Italian neurosurgeons (Zaed et al., 2022). Ethical approval was waived by the local ethics committee in view of the nature of the study.

### 2.2 Survey distribution and administration

The Young Neurosurgeon Committee (YNC) of the EANS administered the survey from April 12 to July 31, 2023. Distribution was carried out via email and social media channels within the EANS community using the Surveymonkey platform (https://www.surveymonkey.com). Data collection occurred online, and the responses were exported into an Excel spreadsheet. Importantly, all responses were provided anonymously.

### 2.3 Survey content

The survey consisted of three different parts: demographics (sex, age, level of education, and place of work); Clance Imposter Phenomenon Survey (CIPS); and analysis of potential compensatory mechanisms (participation in congresses, number of hours worked). CIPS is a cross-culturally validated survey that consists of a 20-item list used to search for imposter characteristics. Responses to each statement are scored along a 5-point scale reflecting the degree of imposter-like feelings ranging from 20 to 100, with a total score of ≤40 indicating few imposter characteristics; 41–60 moderate IS; 61–80 frequent IS; and >80 intense IS feelings. All the subjects who scored a value > 40 on the CIPS were considered as affected by IS; all subjects scoring >60 on the CIPS were considered as frequent/intense IS.

### 2.4 Statistical analysis

The Excel spreadsheet with the raw survey data was imported into the Stata statical analysis program, Version 14.2 for Mac (StataCorp LP, College Station, TX, USA). Count data were reported as frequencies and percentages. We calculated median CIPS scores and interquartile ranges (IQR). Chi-square, univariate analysis of variance (ANOVA) and rank-sum tests were used to examine associations of certain variables (e.g., level of experience, age, board-certification, sex, etc.) with scores on the CIPS, as appropriate. Moreover, univariable and multivariable logistic regression models were built to estimate the likelihood of neurosurgeons with certain characteristics (e.g. female vs. male) to experience frequent/intense IS (CIPS >60 points), reporting (adjusted) odds ratios (OR) and the 95% confidence interval (CI). For statistical analysis, residents in neurosurgery were divided into junior (from the first to the third year) and senior (from the fourth to the last year) residents. Two-sided alpha values of 0.05 were considered statistically significant.

## 3 Results

### 3.1 Demographic data of respondents

We received a total of 232 responses. All demographic data are summarized in Table 1. In summary, most respondents were male (55%) neurosurgeons working at an academic hospital (75%), of which 47% were still in training and 11% in their fellowship. About one out of five had completed a PhD. The main area of interest was well-distributed, with neuro-oncology (27.6%), neurovascular (21.1%) and skull-base (16.4%) chosen most frequently.

**Table 1:** Demographic data of the interviewed population.

	No.	%
**Total respondents**	**232**	100
**Gender**		
Female	105	45.26%
Male	127	54.74%
**Age**		
20–25	12	5.17%
26–30	54	23.28%
31–35	77	33.19%
36–40	38	16.38%
>40	51	21.98%
**Level of education**		
Junior resident	55	23.71%
Senior resident	55	23.71%
Fellow	26	11.21%
Attending	96	41.38%
**Place of work**		
Academic hospital	174	75.00%
Local/Regional Hospital	51	21.98%
Private practice	7	3.02%
**Do you have a PhD**		
Yes	54	23.28%
No	129	55.60%
I am currently doing it	49	21.12%
**Subspecialty of interest**		
Paediatric neurosurgery	24	10.34%
Neuro-oncology	64	27.59%
Functional neurosurgery	15	6.47%
Neurovascular surgery	49	21.12%
Neurotraumatology	11	4.74%
Skull-base surgery	38	16.38%
No particular interest in any subspecialty	31	13.36%
**Working hours per week**		
40–60	51	21.98%
61–80	111	47.84%
81–100	54	23.28%
>100	16	6.90%
**Scientific articles per week**		
0–1	80	34.48%
2–5	119	51.29%
>5	33	14.22%
**Congresses/courses per year**		
0–1	39	16.81%
2–5	162	69.83%
>5	31	13.36%

### 3.2 Prevalence of imposterism among neurosurgeons

According to this definition of IS, a total of 218 respondents (93.97%) had moderate, frequent or intense signs of the syndrome. All data on prevalence are summarized in Table 2. Among the respondents who showed signs of IS, most of them showed moderate (36.21%) or frequent (40.52%) signs; only a minority had an intense symptomatology (17.24%).

**Table 2:** Prevalence of IS and its symptomatology within the interviewed population.

	Total No. (%)	No. of Neurosurgeons/Fellows (%)	No. of Residents (%)
Impostor syndrome			
Present	218 (93.97)		
Not Present	14 (6.03)		
**Impostor symptoms**			
Few	14 (6.03)	10 (8.19)	4 (3.63)
Moderate	84 (36.21)	47 (38.52)	37 (33.63)
Frequent	94 (40.52)	49 (40.16)	45 (40.90)
Intense	40 (17.24)	16 (13.11)	24 (21.81)

Because only a minority of respondents showed few signs of IS, the number was too small to make meaningful statistical analysis between respondents that showed IS versus those who did not. Hence, we focused the statistical analysis on those who showed few or moderate (42.24%) versus frequent or intense (57.76%) signs of IS.

### 3.3 Association of IS with the level of experience/board-certification

Board-certified neurosurgeons, fellows and residents in neurosurgery were considered separately, to see whether there was any difference between the groups. The median CIPS values were 66 for junior residents (IQR 24), 70 for senior residents (IQR 26), 65 for fellows (IQR 22) and 61 for board-certified neurosurgeons (IQR 24); the difference in distribution of CIPS values across the level of experience being close to significant (p = 0.053).

The median CIPS values of board-certified neurosurgeons were 61 (IQR 24) and of residents/fellows were 67 (IQR 25; p = 0.015). Board-certified neurosurgeons were 67.3% as likely as residents/fellows to show frequent/intense IS (OR 0.67, 95% CI 0.39–1.14, p = 0.142).

The median CIPS values of junior residents were 66 (IQR 24) and of senior residents were 70 (IQR 26; p = 0.277). Senior residents were 148% as likely as junior residents to show frequent/intense IS (OR 1.48, 95% CI 0.68–3.21, p = 0.325).

### 3.4 Association of IS with sex

Few, moderate, frequent, and intense IS severity was observed in 2 (1.90%), 31 (29.52%), 45 (42.86%) and 27 (25.71%) of females and in 12 (9.45%), 53 (41.73%), 49 (38.58%) and 13 (10.24%) of male participants (p = 0.001). The median CIPS values of females and males were 69 (IQR 25) and 60 (IQR 23), respectively (p = 0.002). Male neurosurgeons were 44% as likely as female neurosurgeons to show frequent/intense IS (OR 0.44, 95% CI 0.25–0.75, p = 0.003).

### 3.5 Association of IS with other variables

In terms of hospital type, neurosurgeons working in academic, non-academic public or private hospitals had similar scores in the CIPS (p = 0.773). Also, the rates of frequent/intense IS were similar with n = 104 (59.8%) working in academic, n = 26 (51.0%) working in non-academic public, and n = 4 (57.1%) working in private hospitals (p = 0.535). In total, our cohort comprised n = 54 neurosurgeons with a PhD title, and n = 129 without. Neurosurgeons with a PhD title were 57% as likely as those without a PhD title to show frequent/intense IS (OR 0.57, 95% CI 0.30–1.08, p = 0.085).

### 3.6 Compensatory mechanisms for IS

In order to see, whether the people with imposterism were more prone to compensate for their feeling of guilt, the respondents were asked about their working time. The amount of working hours/week was not associated with the CIPS score (p = 0.222) and the rates of frequent/intense IS were similar between neurosurgeons working 40–60 h (n = 24; 47.06%), 61–80 h (n = 67; 60.36%), 81–100 h (n = 32; 59.26%), or >100 h (n = 11; 68.75%; p = 0.315).

As suggested in the existing literature, gathering data about the relationship between IS and clinical competence could be of importance. To analyze this relationship, we chose to evaluate the number of papers read per week by our respondents, although there is no direct relationship between this number and clinical competence. This number was also not associated with the CIPS score (p = 0.981) and the rates of frequent/intense IS were similar between neurosurgeons reading 0–1 article (n = 44; 55.00%), 2–5 articles (n = 69; 57.98%), or >5 articles (n = 21; 63.64%; p = 0.698).

Participation in congresses and formation courses was also investigated. Similarly, the number of congresses/courses attended annually was not associated with the CIPS score (p = 0.870) and the rates of frequent/intense IS were similar between neurosurgeons attending 0–1 event (n = 23; 58.97%), 2–5 events (n = 92; 56.79%), or >5 events (n = 19; 61.29%; p = 0.885).

#### 3.6.1 Multivariable logistic regression model

To account for potential confounding factors, a multivariable model was built to estimate the likelihood for frequent/intense IS, including the dichotomized variables sex, age >35 years, achievement of board-certification, achievement of PhD title, academic hospital type, working >80 h per week, reading >5 articles per week and attending >5 congresses/courses per year (Table 3). In both the uni- and multivariable model, male sex emerged as an independent protective factor for frequent/intense IS (adjusted OR 0.43, 95% CI 0.22–0.83, p = 0.012). Reading >5 articles per week was not associated with frequent/intense IS in the univariable model but was identified as an independent risk factor for frequent/intense IS in the multivariable model. Neurosurgeons reading >5 articles per week were more than three times as likely as those reading less to experience frequent/intense IS (adjusted OR 3.03, 95% CI 1.03–8.94, p = 0.045).

**Table 3:** Uni- and multivariable logistic regression model, estimating the likelihood to show frequent/intense IS (CIPS scores of >60). Results are expressed as (adjusted) odds ratios (OR) with 95% confidence intervals (CIs).

Determinants	Univariate analysis	Multivariate analysis
Male sex	0.44, 0.25–0.75, p = 0.003	0.43, 0.23–0.83, p = 0.012
Age >35 years	0.67, 0.39–1.14, p = 0.140	0.80, 0.34–1.90, p = 0.617
Board-certification	0.67, 0.40–1.14, p = 0.142	0.87, 0.38–2.00, p = 0.744
PhD title	0.57, 0.30–1.08, p = 0.085	0.61, 0.28–1.31, p = 0.204
Academic hospital	1.38, 0.76–2.52, p = 0.284	1.34, 0.66–2.71, p = 0.412
Working >80 h/week	1.24, 0.70–2.20, p = 0.457	0.72, 0.35–1.45, p = 0.353
Reading >5 articles/week	1.33, 0.62–2.85, p = 0.461	3.03, 1.03–8.94, p = 0.045
Participate in >5 congresses/year	1.18, 0.54–2.57, p = 0.669	1.03, 0.40–2.66, p = 0.950

## 4 Discussion

In this cross-sectional study of 232 neurosurgeons and residents in neurosurgery all over Europe, the majority of participants experienced frequent imposter-like feelings, as the previous study conducted on the neurosurgical community part of the EANS (Zaed et al., 2022). Several studies in different surgical specialities have already highlighted that there is no significant association between the degree of IS and the ethnicity/race of the clinician or the region of the country of residence (Addae-Konadu et al). However, we found a negative correlation between the degree of imposterism and the level of medical experience, in that more experienced clinicians reported symptoms significantly less frequently. These results are similar to a previous study, where neurosurgeons practicing for years had gained increasing confidence, which resulted in a lower degree of IS (Zaed et al., 2022). We assumed that some characteristics could turn out to be correlated with the presence or level of imposterism.

When analyzing factors like surgeons’ gender, stage of training, and academic achievements (in this case a PhD degree), we found strong correlation between gender and IS, as well as academic degree, with female gender and PhD holders being respectively more and less prone to be affected by IS. Interestingly, this statistical difference was also retained when we compared these two factors (sex and PhD) with the severity of symptoms. This finding confirm the conclusions of a recent article that suggest that the gender features influence behaviour in workplaces like neurosurgery. Somma et al. found that women are more prone to be affected by IS because they tend to express their own fears and point out their shortcomings, thus underrating themselves (Somma and Cappabianca, 2019). As for the general level of education, not only a difference was documented in the prevalence of IS between residents and young neurosurgeons, numbers differ greatly also in the severity of symptoms, with residents showing a higher frequency of more severe symptoms. When junior residents were analyzed separately, no statistical difference was found in terms of prevalence and severity of symptoms.

Our results are in line with the recent literature. Oriel et al. found that IS was more prevalent among female residents, and individuals with IS were more likely to experience depression, anxiety, and low self-esteem (Oriel et al.). In another interesting pilot study in which the level of burnout and IS in medical students at Jefferson Medical College was discussed, Villwock et al. found that IS was more prevalent among female medical students (Villwock et al). There was a significant positive trend between race/ethnicity and imposter feelings as members of a minority experienced IS more frequently compared to their majority counterparts. IS was associated with burnout, cynicism, emotional exhaustion, and depersonalization (Villwock et al). Based on their findings, the investigators concluded that there were potential implications of IS in medical training; e.g., students who experience IS may be less likely to speak up or volunteer answers than unaffected peers. Individuals with IS may have innate differences in learning styles. The investigators urged educators to consider tailoring medical education curricula to accommodate learners with IS (Clance, 1985).

Recently an article by Hanjani et al. (Amin-Hanjani and Haglund, 2022) highlighted that the alternative of IS could be worse. Insofar, as IS signifies excessive doubt in one's abilities, the opposite end of the spectrum (i.e., excessive confidence) can be equally or even more damaging in the context of patient care. Both excessive doubt and overconfidence carry risks. Ultimately, as with many psychosocial behaviours, moderation is likely to be warranted, and different situations may call for differing degrees of confidence.

The correlation between IS and burnout syndrome should also be further investigated (Clance, 1985; Zaed et al.; Menna and Zaed, 2021). Neurosurgery remains the medical speciality with the highest rate of burnout, not only among board certified neurosurgeons but also among residents in neurosurgery (Zaed et al., 2022). The prevalence of IS, especially in a community of young neurosurgeons, could indeed represent a significant burden in terms of psychological impact throughout training extending to the first years of clinical practice, given that it can affect young neurosurgeons’ self-confidence and the awareness of personal strengths and limitations.

Moving forward, research should delve into external factors contributing to IS development, while devising strategies to mitigate its impact. Interventions such as mentorship programs, mental health resources, and resilience-focused educational initiatives tailored to the unique demands of neurosurgery could be instrumental. Also group and individual interventions such as workshops, small group discussions and coaching can be used to overcome IP in healthcare (Siddiqui et al., 2024).

Future research should focus on identifying how external factors may contribute to the development of IS, and additional strategies should be conceived to mitigate its impact. Maybe the academic environments themselves may inherently scale down the self-perceptions of residents. Perhaps being part of a highly competitive environment devoted to the perpetual progress of medical science might turn out to be an incessant reminder of what is yet to be known, discovered, or improved.

### 4.1 Study limitations

This study presents several noteworthy limitations. Firstly, the cross-sectional design employed does not facilitate the exploration of causal relationships between variables. As such, it fails to capture the dynamic interplay and temporal sequences that underlie phenomena. Furthermore, the inherent nature of survey methodologies introduces the well-documented “Hawthorne effect.” This phenomenon, characterized by altered behavior due to the awareness of being observed, poses a significant source of bias in studies of this nature. The reliance on self-reported data in this project, while ensuring respondent anonymity, inherently renders the results subjective to each neurosurgery resident's perspective. Personal factors such as stress, frustration, and other situational contingencies may have influenced respondents' answers, potentially leading to variability in responses across different occasions. Although the study achieved a commendable response rate, potential survey response bias must be acknowledged. The disproportionate representation of respondents from academic institutions and young residents suggests a sample bias that could skew the prevalence rates reported. Moreover, the predominance of young residents and those from academic backgrounds may render them more sensitized to the issue under investigation, thereby affecting the overall prevalence rates. Additionally, the literature indicates that hierarchical structures in medical education, particularly in academic settings, may exacerbate feelings of Imposter Syndrome (IS). However, it is noteworthy that academic environments also provide conducive settings for organizing workshops on IS and developing mentorship and support programs, which have shown promise in mitigating IS-related symptoms. Given the cross-sectional nature of the survey, its capacity to capture the longitudinal evolution of IS symptoms over a neurosurgeon's career trajectory is limited. Acknowledging this constraint, future research endeavors could explore longitudinal studies to provide a more comprehensive understanding of IS dynamics over time. Furthermore, the potential for response bias, where individuals experiencing IS may be more inclined to participate in a survey about the syndrome, warrants critical examination. This self-selection bias has the potential to skew reported prevalence rates and should be addressed in future research to ensure the robustness of findings. (Bhama et al., 2021).

## 5 Conclusion

Frequent or intense signs of IS appears to be highly prevalent in the neurosurgical population studied with 57,8% respondents reporting potentially significant levels.

This survey identified female gender associated with a significantly higher rate of IS in neurosurgery, and academic environment as a possible protective factor. The implications of IS on both the quality of care for patients and the well-being of neurosurgeons should be evaluated in future studies, with a greater focus on subspecialties and career stages.

## Disclosure of potential conflict of interest

The authors report no conflict of interest concerning the materials or methods used in this study or the findings specified in this paper.

## Author contributions

Concept and design: Zaed, Zoia. Acquisition of data: All authors. Analysis and interpretation of data: All authors. Drafting the article: Zaed, Zoia, Stienen. Critically revising the article: All authors.

## Consent

All partecipants have given written consent for publication of this study.

## Funding

The authors declare that the article content was composed in the absence of any commercial or financial relationships that could be construed as a potential conflict of interest.

## Declaration of competing interest

The authors declare that they have no known competing financial interests or personal relationships that could have appeared to influence the work reported in this paper.
